# OLDER ADULTS DURING THE COVID-19 PANDEMIC: ROLE OF HEALTH CONDITIONS IN RESILIENCY AND PERCEIVED STRESS

**DOI:** 10.1093/geroni/igac059.1954

**Published:** 2022-12-20

**Authors:** Allyson Sloan

**Affiliations:** Johns Hopkins University School of Medicine, Baltimore, Maryland, United States

## Abstract

The COVID-19 pandemic has necessitated protracted lockdowns, exacerbating the challenges associated with social isolation. Older adults, who often suffer from social isolation, may have a harder time coping with the added isolation imposed by COVID-19. In this study, we investigated the resiliency and coping skills of older adults amidst the COVID-19 pandemic, and whether they were associated with different co-morbidities. We conducted a 45-minute telephone survey of 107 participants to assess their experiences during the COVID-19 pandemic. Participants were recruited from existing Johns Hopkins studies. The survey included the Brief Resilient Coping Scale, the Perceived Stress Scale, and questions about current health conditions (arthritis, osteoporosis, high blood pressure, heart disease, and depression/anxiety) and whether these conditions worsened, improved, or remained unchanged since the beginning of the pandemic. We regressed scores for resiliency and coping scores on a series of indicators of whether co-morbidities had worsened during the pandemic. On average, participants were 76 years old and 63% were female. There was no association between resiliency scores and any of the co-morbidities (r2=2.8%). With respect to perceived stress, participants who reported their depression/anxiety worsened during the pandemic also reported greater levels of perceived stress (B=0.83, 95% confidence interval: 0.30, 1.36). We were surprised that additional co-morbidities did not affect resiliency or stress. In order to better serve geriatric populations, health professionals should closely monitor those patients who have depression and anxiety during times of social distancing, epidemics, or pandemics.

